# Effects of parental smoking and indoor tobacco smoke exposure on respiratory outcomes in children

**DOI:** 10.1038/s41598-020-60700-4

**Published:** 2020-03-09

**Authors:** Yang Zhuge, Hua Qian, Xiaohong Zheng, Chen Huang, Yinping Zhang, Baizhan Li, Zhuohui Zhao, Qihong Deng, Xu Yang, Yuexia Sun, Xin Zhang, Jan Sundell

**Affiliations:** 1 School of Energy and Environment, Southeast University, Nanjing, China; 2 School of Environment and Architecture, University of Shanghai for Science and Technology, Shanghai, China; 3 Beijing Key Lab of Indoor Air Quality Evaluation and Control, Tsinghua University, Beijing, China; 4 Key Laboratory of Three Gorges Reservoir Region’s Eco-Environment, Chongqing University, Chongqing, China; 5 School of Public Health, Fudan University, Shanghai, China; 6 School of Energy Science and Engineering, Central South University, Changsha, Hunan China; 7 College of Life Science, Central China Normal University, Wuhan, China; 8 School of Environmental Science and Engineering, Tianjin University, Tianjin, China; 9 Institute of Environmental Science, Shanxi University, Taiyuan, China

**Keywords:** Environmental impact, Public health, Risk factors

## Abstract

The extensive literature has reported adverse effects on environmental tobacco smoke (ETS) on children’s health. We aim to analyze associations of ETS with dry night cough, croup, pneumonia, and frequent common cold and to disentangle the effects of prenatal, infancy and childhood exposure by multilevel logistic regression. A cross-sectional study was conducted among 41,176 children aged 3–8 years in 8 major cities of China during 2010–2011, and obtained demographic information, smoke exposure information, and respiratory outcomes. Parents’ smoking habit and indoor tobacco smoke odor were considered as two indicators of ETS. The prevalences of respiratory outcomes were 6.0% for croup, 9.5% for frequency common cold, 17.1% for dry night cough and 32.3% for pneumonia respectively in the study. The associations between respiratory outcomes and parental smoking were not obvious, while indoor tobacco smoke odor was clearly and strongly associated with most respiratory outcomes, with adjusted odds ratios ranging from 1.06 to 1.95. Both infancy and childhood exposure to tobacco smoke odor were independent risk factors, but infancy exposure had a higher risk. The results explore that ETS increased the risk of respiratory outcomes in children, highlighting the need for raising awareness about the detrimental effects of tobacco smoke exposure.

## Introduction

Conclusive evidence demonstrates the detrimental effects of environmental tobacco smoke (ETS) on respiratory symptoms, including increasing the incidence of asthma^1–3^, wheeze^3,4^, rhinitis^5^, lower and upper respiratory infections^6–8^ in children. ETS consists of more than 4000 components, of which more than 40 are carcinogens^9^. WHO (World Health Organization) has estimated that about half of the world’s children, ~700 million have been exposed to tobacco smoke, mainly in their homes^10^. Children are more vulnerable to ambient air pollutants than adults as their immune systems have not been well developed^11^. Respiratory tract infections (RTI) are the predominant causes of mortality and morbidity among children^12^. RTIs are traditionally divided into upper respiratory tract infections (such as common cold) and lower respiratory tract infections (such as pneumonia). Although abundant studies support causal associations between tobacco smoke exposure and respiratory outcomes among children^6,13–15^, associations are inconclusive, perhaps because of differences in the extent of tobacco smoke exposure, questionnaires, populations and sample size across studies.

Several studies focused on the independent effect of prenatal, postnatal and childhood exposure to tobacco smoke on children’s respiratory outcomes^1,16–18^, yielding inconsistent findings. A study in six cities of metropolitan France of 9000 children aged 9–11 years old found there were no associations between parental smoking and atopy, rhinitis, eczema^19^. A study based on the first 22,390 children born between 2000 and 2004 in the Norwegian Mother and Child Cohort found postnatal paternal smoking was also associated with these outcomes, independently of maternal smoking in pregnancy^20^. It was difficult to distinguish the effects of prenatal exposure with those of postnatal exposure on children. Smoking during pregnancy is likely to post an additional risk besides postnatal exposure to ETS^16^. Maternal smoking has greater detrimental effects than paternal smoking on the respiratory health of children and maternal smoking during pregnancy has been associated with adverse respiratory outcomes in children^21^.

Although some international studies have provided some important data on the relationship between smoke exposure and children’s respiratory health, children’s health problems in China have not drawn sufficient attention and the health impact of tobacco smoke exposure has not been well studied. A large cross-sectional survey of 8 major cities of mainland China has been conducted among 41,176 children. Demographic data, smoke exposure information and respiratory outcomes of children were collected via questionnaires. The main aim of the present study is to assess children’s exposure to tobacco smoke in utero, in the first year of life and childhood as risk factors for respiratory outcomes (including pneumonia, common cold, croup, and dry night cough). Our hypothesis is that earlier exposure to tobacco smoke exerts greater effects and that tobacco smoke exposure in any individual period is an independent risk factor for investigated respiratory outcomes.

## Methods

### Study design and population

**Figure 1:**
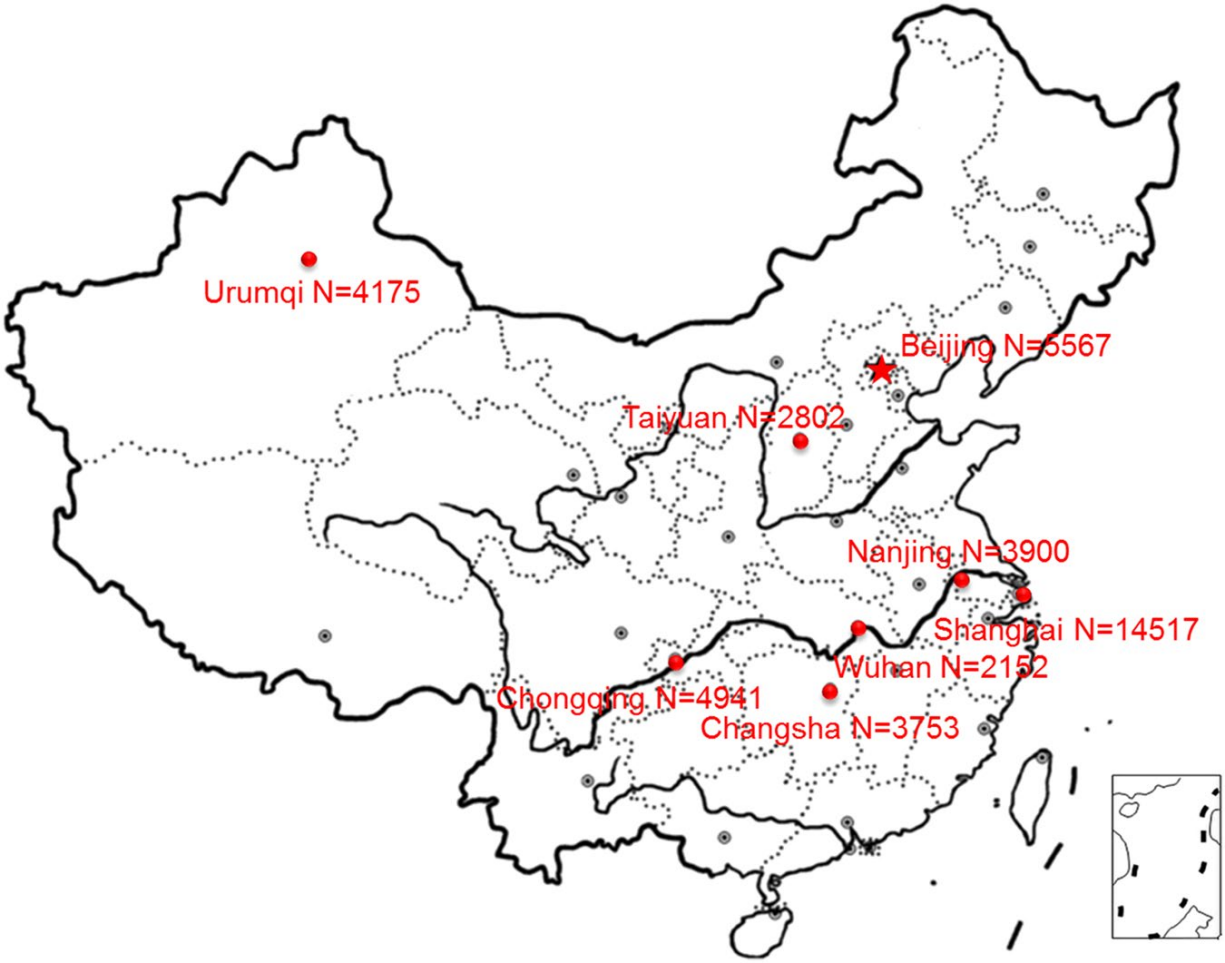
Map of sample sizes in eight Chinese cities.

This research is a part of the Children, China, Homes, Health (CCHH) project, a national study of childhood respiratory outcomes and home environments^22,23^. The study was conducted in more than 200 kindergartens in 8 metropolitan cities of China (Beijing, Taiyuan, Urumqi, Wuhan, Changsha, Shanghai, Nanjing, and Chongqing) respectively from January 2010 to December 2012. The study population consisted of pre-school children aged 1–8 years. Questionnaires were randomly distributed to children in >200 kindergartens and were filled out by the children’s parents or other guardians and then returned to teachers of kindergartens. Meanwhile, children’s parents or other guardians were informed that questionnaire data would be used for scientific research and consent had been obtained from them. The sample size of each city is shown in Fig. 1. Questionnaires were distributed in kindergartens, filled out by children’s parents or other guardians and returned to teachers of kindergartens within one week.

### Health outcomes and exposure assessment

Responses to the questionnaires yielded demographic information, health outcomes, environmental exposure, and living habits. The health questions were adopted from the ISAAC (International Study of Asthma and Allergies in Childhood) studies^24^. Questions on environmental factors and lifestyles were similar to those in studies from Sweden^25^, Bulgaria^26^, but were modified to characterize Chinese building characteristics. We analyzed the health outcomes in Table 1, the incidences represented the proportion of children who had ever had the disease or the symptom. We investigated parental smoking and tobacco odor in the residence (judged by respondents) using the questions in Table 2.

**Table 1:** Description and questions related to investigated respiratory outcomes.

Symptoms	Questions
Dry night cough	In the last 12 months, has your child had a dry cough at night for more than two weeks, apart from a cough associated with a cold or chest infection? Yes/no.
Croup	Has your child had croup (breathing difficulties with dry cough)? Yes/no.
Pneumonia	Has your child been diagnosed with pneumonia by a doctor? Yes/no.
Frequency common cold	In the past 12 months, how many times has your child had a cold? <6 times/≥6 times

**Table 2:** Definitions and questions related to tobacco smoke exposure.

When	Category	Questions
In utero (during pregnancy)	Parental smoking	Did the child’s parents smoke during the pregnancy? (both/mother/father/neither)
The first year (during infancy)	Parental smoking	Did the child’s parents smoke when the child was born? (both/mother/father/neither)
	Tobacco odor	Did you notice tobacco odor in your home when the child was born? (yes, frequently/sometimes/never)
Current (during childhood)	Parental smoking	Do the child’s parents smoke now? (both/mother/father/neither)
	Tobacco odor	Do you notice tobacco odor in your home in recent 3 months? (yes, frequently/sometimes/never)

### Statistical analysis

Multilevel regression was used to assess associations between respiratory outcomes and smoke exposure, and associations were adjusted for children’s demographic data and environmental risk factors. Two levels were used (city and child). Demographic data contain age, sex, breastfeeding duration, birth weight, and family allergic history. Environmental factors include the region of the current residence, building type, dwelling areas, home dampness exposure, cooking fuel type, redecoration or new furniture, cockroaches noted and living habits (cleaning child’s bedroom and putting bedding to the sunshine). The percentage of croup and frequent common cold were rare (<15%) which needs a multilevel Poisson regression model. The percentage of pneumonia and dry night cough were rare (>15%) which needs a multilevel logistic regression model. Odds ratios (ORs) were calculated with a 95% confidence interval. We accepted significance for *P*-values less than 0.05. SPSS11.0 and STATA 15.1 was used to perform statistical analyses.

### Ethics statement

The project was approved by the ethical committee of the School of Public Health, Fudan University in Shanghai, China (International Registered Number: IRB00002408&FW A00002399). All experiments were performed in accordance with relevant guidelines and regulations. Informed consent was obtained from the legal guardians of children.

## Results

A total of 44,859 completed questionnaires were received. After the exclusion of 2989 questionnaires for missing sex and age data, 41,176 valid questionnaires were received. The study was restricted to 3–8 years old children because infancy and childhood exposure was difficult to distinguish for 1–2 years children and their sample sizes were relatively small.

Table 3 presents demographic data, frequency of respiratory outcomes and exposure to tobacco smoke. The mean age of the investigated children is 4.65 years. Boys accounted for 51.9% and girls for 48.1%. Among respiratory outcomes, pneumonia was reported most frequently, with a 32.3% lifetime-ever incidence in the investigated children. Croup was reported least frequently with a lifetime-ever incidence of 6.0%. The incidence of common cold more than 6 times in recent 12 months was reported by 9.5%, while 17.1% reported having had dry night cough for more than two weeks and 14.3% of children had at least one respiratory symptom. About 45% of parents were smokers, but smoking mothers accounted for less than 1.0%. Of investigated children, 42.9% were exposed to parental smoking during pregnancy, 45.4% during the first year of life and 45.4% were currently exposed. Indoor tobacco odor was reported by 20.9% during the first year of life, and 29.2% currently.

**Table 3:** Demographic data, frequency of respiratory outcomes and exposure to tobacco smoke.

Category	Sample size n	%
Age	Mean 4.65 ± 1.12	
Sex	Male 21375 (51.9%)	Female 19801 (48.1%)
**Disease**		
Dry night cough	6991	17.1
Croup	2436	6.0
Pneumonia	13300	32.3
Frequent common cold	3726	9.5
Number of diseases		
0	20246	52.7
1	12658	33.0
2	4294	11.2
3	1049	2.7
4	142	0.4

Table 4 presents the respiratory outcomes stratified by children’s demographic data and environmental exposures. Generally, incidences were greater in younger children, boys, children with low birth weight, children whose breastfeeding duration was less than 6 months, and children with a family history of atopy. Most of the differences were statistically significant. Environmental factors were also associated with elevated incidences. Specifically, home dampness exposure, solid or natural gas cooking fuel, residence redecoration or new furniture were associated with increased incidences of respiratory outcomes. More, several living habits like cockroaches or rats noted and burning mosquito coils also resulted in higher incidences but putting bedding to the sunshine frequently and the cleaning child’s bedroom daily were inverse.

**Table 4:** Respiratory outcomes stratified by demographic data and environmental exposures.

Categories	No. of subjects	Dry night cough (%)	Croup (%)	Pneumonia (%)	Frequent common cold (%)
**Demographic data**					
Age					
3 years	6911	19.9	5.0	30.5	12.1
4 years	13523	18.3	6.2	33.3	11.0
5 years	11723	16.3	6.5	33.5	8.5
6 years	6273	14.4	5.9	33.5	6.7
7 years	1612	14.4	6.4	26.4	5.9
8 years	1134	12.9	5.8	20.9	4.9
Sex					
Male	21375	17.0	7.0	33.8	9.7
Female	19801	17.2	5.0	30.7	9.2
Breastfeeding duration					
>6 months	20939	15.7	5.5	29.6	9.3
≤6 months	19349	18.8	6.6	35.3	9.7
Family allergic history					
Yes	7960	26.1	11.3	42.2	13.3
No	30420	15.1	4.8	30.4	8.7
Birthweight					
<2500 g	1054	17.2	7.8	38.4	10.8
≥2500 g	39083	17.1	6.0	32.2	9.4
**Environmental factors**					
Region of the current residence					
Urban	30400	17.4	6.1	33.6	9.1
Suburban/rural	9828	16.3	5.8	29.1	10.6
Home dampness exposure					
Yes	12725	18.8	7.0	34.6	10.0
No	9992	12.5	4.4	29.7	7.5
Cooking fuel type					
Solid fuel/natural gas	28712	17.8	6.2	33.3	9.3
Electricity	10058	15.9	6.0	29.4	9.9
Redecoration or new furniture					
Yes	24754	18.8	6.5	33.7	10.0
No	8949	15.1	5.7	31.9	9.2
Cockroaches noted					
Yes	20722	18.5	7.1	33.4	10.3
No	14919	15.1	5.1	31.8	8.2
Cleaning child’s bedroom daily					
Yes	21990	16.2	5.7	31.6	8.6
No	18091	18.1	6.5	33.6	10.3
Putting bedding to the sunshine frequently					
Yes	25654	16.7	5.7	31.1	8.4
No	14806	17.9	6.6	34.5	11.0
**Smoke exposure**					
Parental smoking during pregnancy					
Yes	16436	17.9	6.6	33.5	9.8
No	22267	17.5	5.8	31.7	9.6
Parental smoking during infancy					
Yes	17807	17.5	6.5	33.3	9.6
No	21477	17.5	5.8	31.7	9.6
Parental smoking during childhood					
Yes	18873	17.1	6.4	33.1	9.6
No	22357	17.2	5.7	31.7	9.2
Tobacco odor indoors during infancy					
Frequently	1260	27.4	11.3	39.4	13.8
Sometimes	7327	19.3	7.6	33.8	10.5
Never	25287	15.6	5.5	32.2	8.7
Tobacco odor indoors during childhood					
Frequently	2154	24.1	9.3	37.9	13.1
Sometimes	9871	18.9	7.0	33.3	9.9
Never	22028	15.2	5.4	32.1	8.6

Sample sizes are not 41,176 due to missing data.

Table 5 compares the effects of maternal smoking and father smoking. The maternal smoking rate in China is much lower than the paternal smoking rate. The associations between maternal smoking and investigated outcomes were weak. While father smoking was stronger associated with most respiratory health outcomes, except for the frequent common cold. Mother and father both smoked also had week effects on outcomes.

**Table 5:** Crude odds ratios(95% CI) for respiratory health outcomes associated with parental smoking by using two-level regression model.

	No. of subjects	Dry night cough	Croup	Pneumonia	Frequent common cold
During pregnancy					
Neither	22267	1	1	1	1
Mother only	148	1.22(0.82–1.84)	1.10(0.58–2.10)	0.97(0.69–1.38)	1.01(0.58–1.76)
Father only	16230	1.06(1.00–1.12)*	1.16(1.07–1.26)*	1.08(1.03–1.12)*	1.01(0.94–1.08)
Both	58	1.44(0.79–2.63)	1.25(0.45–3.48)	0.93(0.53–1.64)	0.44(0.14–1.42)
During infancy					
Neither	21477	1	1	1	1
Mother only	193	1.12(0.78–1.63)	1.32(0.77–2.24)	0.86(0.63–1.17)	0.85(0.50–1.44)
Father only	17524	1.03(0.97–1.08)	1.14(1.05–1.24)*	1.05(1.01–1.10)*	0.98(0.91–1.05)
Both	90	1.68(1.05–2.69)*	1.67(0.80–3.46)	0.95(0.60–1.50)	0.79(0.38–1.64)
During childhood					
Neither	22357	1	1	1	1
Mother only	288	1.39(1.05–1.84)	1.21(0.77–1.92)	0.88(0.68–1.14)	0.82(0.53–1.27)
Father only	18340	1.02(0.96–1.07)	1.14(1.05–1.24)*	1.05(1.01–1.10)*	0.93(0.87–1.00)
Both	245	1.25(0.92–1.71)	1.21(0.73–1.98)	0.97(0.74–1.28)	0.86(0.55–1.36)

*P-value < 0.05.

Table 6 presents crude odds ratios for respiratory health outcomes as associated with ETS. Parental smoking (mother or paternal smoking) was associated with investigated respiratory outcomes significantly, except for the frequent common cold. Moreover, the ORs for the associations were similar among pregnancy, infancy and childhood exposure. Indoor tobacco smoke odor was also associated with investigated respiratory outcomes significantly, except for dry night cough during childhood.

**Table 6:** Crude odds ratios(95% CI) for respiratory health outcomes associated with ETS by using two-level regression model.

		Dry night cough	Croup	Pneumonia	Frequent common cold
**Parental smoking**					
Pregnancy exposure	Yes/no	1.06(1.01–1.12)*	1.16(1.07–1.26)*	1.07(1.03–1.12)*	1.00(0.94–1.08)
During infancy	Yes/no	1.03(0.97–1.09)*	1.15(1.05–1.25)*	1.05(1.01–1.10)*	0.98(0.91–1.05)
During childhood	Yes/no	1.06(1.00–1.12)*	1.15(1.06–1.26)*	1.07(1.02–1.12)*	0.99(0.92–1.06)
**Tobacco odor indoors**					
During infancy	Sometimes/never	1.24(1.15–1.33)*	1.46(1.31–1.63)*	1.13(1.07–1.20)*	1.16(1.05–1.27)*
	Frequently/never	2.03(1.77–2.33)*	2.31(1.91–2.79)*	1.39(1.23–1.57)*	1.64(1.37–1.95)*
During childhood	Sometimes/never	1.22(1.15–1.31)	1.37(1.24–1.51)*	1.11(1.05–1.17)*	1.11(1.02–1.21)*
	Frequently/never	1.72(1.53–1.93)	1.89(1.61–2.23)*	1.33(1.21–1.47)*	1.54(1.34–1.78)*

*P-value < 0.05.

Table 7 presents the adjusted odds ratios for respiratory health outcomes as associated with ETS. After adjustment, most associations between parental smoking and outcomes were insignificant, while most associations between tobacco odor and outcomes remained robust. Frequently perceived tobacco odor had the greatest AOR value than any of the less frequent for all outcomes, both during infancy or childhood. Higher AOR values were observed for croup and dry night cough than pneumonia or a frequent common cold. Dry night cough was the outcome most strongly associated with indoor tobacco odor, with the highest AOR 1.95 for frequent exposure during infancy.

**Table 7:** Adjusted odds ratios(95% CI) for respiratory health outcomes associated with ETS by using two-level regression model.

		Dry night cough	Croup	Pneumonia	Frequent common cold
**Parental smoking**					
Pregnancy exposure	Yes/no	1,03(0.95–1.13)	1.13(0.98–1.29)	1.04(0.97–1.11)	1.02(0.91–1.14)
During infancy	Yes/no	1.00(0.92–1.10)	1.16(1.01–1.33)*	1.04(0.97–1.11)	0.97(0.87–1.08)
During childhood	Yes/no	1.05(0.95–1.13)	1.19(1.04–1.36)*	1.05(0.98–1.12)	1.00(0.89–1.12)
**Tobacco odor indoors**					
During infancy	Sometimes/never	1.22(1.10–1.36)*	1.37(1.16–1.61)*	1.11(1.01–1.21)*	1.10(0.96–1.26)
	Frequently/never	1.95(1.58–2.41)*	1.89(1.38–2.57)*	1.21(1.00–1.47)*	1.54(1.17–2.02)*
During childhood	Sometimes/never	1.14(1.03–1.26)*	1.32(1.13–1.53)*	1.05(0.97–1.13)	1.06(0.94–1.21)
	Frequently/never	1.56(1.31–1.85)*	1.74(1.36–2.24)*	1.15(0.99–1.33)	1.38(1.11–1.71)*

*P-value < 0.05.

Adjusted for age, sex, low birth weight, breastfeeding duration, family allergic history, region of the current residence, bought new furniture and/or redecorated the residence, dampness exposure, cooking fuel type, cockroaches noted, cleaning daily and putting bedding to the sunshine frequently.

**Figure 2:**
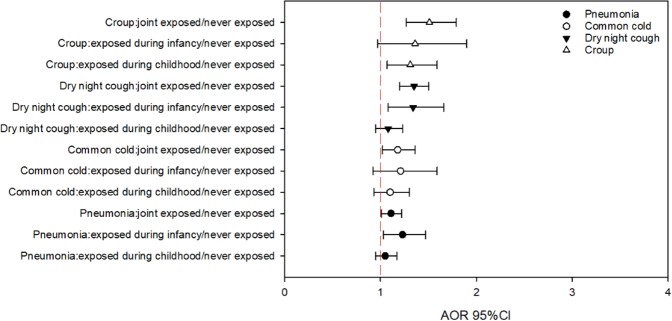
Independent effects of infancy and childhood tobacco smoke exposure on respiratory outcomes (using indoor smoke odor as the indicator). Adjusted for age, sex, low birth weight, breastfeeding duration, family allergic history, region of the current residence, bought new furniture and/or redecorated the residence, dampness exposure, cooking fuel type, cockroaches noted, cleaning daily and putting bedding to the sunshine frequently.

Perceived indoor tobacco odor had a greater association than parental smoking with respiratory diseases. We treated indoor tobacco odor (frequently or sometimes) as an indicator of ETS exposure for children, and then we assessed the independent effects of infancy and childhood exposure on respiratory outcomes (Fig. 2). Children exposed to indoor tobacco odor either during infancy or childhood had increased risk of respiratory outcomes, with infancy exposure resulting in higher relative risks. For pneumonia, exposure during infancy only (AOR 1.23 95% CI 1.03–1.47) had the highest AOR. For croup, exposure during both infancy and childhood (AOR 1.51 95% CI 1.27–1.79) had the highest AOR. For the common cold, exposure during infancy only (AOR 1.21 95% CI 0.92–1.59) had the highest AOR. For dry night cough, exposure during infancy only (AOR 1.34 95% CI 1.08–1.66) and joint exposure had similar AOR (AOR 1.35 95% CI 1.20–1.50).

## Discussion

Previous studies found clear and significant relationships between parental smoking and respiratory outcomes^27–29^. Different from those studies, parental smoking may not accurately indicate children’s ETS exposure in this study. There may be several reasons for this. Most of the investigated families only had one child because of China’s one-child policy, enabling the child to be cherished and well cared for by family members. Moreover, the adverse effects of passive smoking have been well acknowledged among parents in China. Therefore, those parents who smoke may avoid smoking in the presence of children. This is supported by the weak relationship between parental smoking and tobacco smoke odor (correlation coefficient: current 0.369; during pregnancy 0.291). Among residences without current smoking parents, 18.5% had tobacco odor (2.1% frequently and 16.4% sometimes). Among families with smoking parents, 46.0% never perceived smoke in the house, 43.0% perceived smoke sometimes and only 11.0% perceived smoke frequently. The perceived indoor smoke odor could be a more direct indicator of ETS exposure than parental smoking in our study.

Croup is a common cause of upper airway obstruction in infants and young children, which affects more boys than girls^30,31^. Few studies focused on croup and its related risk factors but it is a common cause of upper airway obstruction in infants and young children, which is typically caused by parainfluenza, influenza or other viruses^32^. A study in Finland found that smoking by both parents was associated with a decreased occurrence of both croup and recurrent croup^33^.

Cough is a very common symptom of respiratory tract infections such as the common cold, acute bronchitis, pneumonia, pertussis, or tuberculosis^34^. Nocturnal dry cough, however, is highly indicative of asthma^35^ and can be induced mechanically or chemically by endogenous or exogenous agents, such as particulate matter (PM_2.5_) and nitrogen dioxide (NO_2_)^36^. Our previous study in Nanjing provided the associations between dry cough and sensation of stuffy odor, unpleasant odor, pungent odor, moldy odor, humid air, and dry air (*P* < 0.05)^37^. Environmental tobacco smoke exposure has been reported to be associated with cough^38^, but some studies found no relation between dry night cough and smoke exposure^39,40^. However, a pooled analysis of 53 879 children from 12 cross-sectional studies found strong evidence linking nocturnal cough to smoking during pregnancy, smoking during the first two years and parental current smoking^1^. A study in Manchester reported a statistically significant increase in the risk of night cough for children who lived with smokers in a household (aOR 1.45 95% CI 1.20–1.75)^41^.

The common cold is reported as one of the most common infectious diseases in young children and the frequency of colds declines with advancing age^42^. The common cold is the most frequently infected disease and children usually got common cold for 6–12 times annually^43^. The relationship between the common cold and smoke exposure was not well studied. It is reported that indoor dampness and keeping cats or dogs as pets but not smoke exposure, can be risk factors for common cold^44^.

Pneumonia is a lower respiratory infection caused primarily by bacteria or viruses and was the leading infectious cause of mortality in children under 5 years old in 2015 worldwide^45^. Exposure to parental smoking is one identified pneumonia-related residential risk factor^46^ and several studies have reported environmental tobacco smoke to be a risk for pneumonia in children^47–50^. A study in China reported household exposure to cigarette smoke to be a risk factor for pneumonia in children^51^.

We were able to separately assess the effects of infancy and childhood exposure on children’s respiratory health and found that both were independent risk factors. Infancy-only exposure seemed to have a stronger effect than childhood-only exposure. Most studies focused on the differential effects of prenatal and postnatal tobacco smoke tended to support a stronger effect of prenatal exposure on respiratory symptoms^52,53^. Maternal smoking had an insignificant effect on respiratory outcomes in this paper. Inconsistent with our findings, a great deal of evidence indicated that maternal smoking was strongly related to children’s health outcomes especially during pregnancy^54–56^, but most surveys were conducted in western countries. An explanation for the insignificant association in the present study is that the maternal smoking rate in China is much lower than the paternal smoking rate. WHO in 2010 estimated that about 51% of men and about 2% of women smoke in China^57^. Only a small portion of mothers had the habit of smoking in the present study. The sample was too small to have statistical power.

There are some limitations in this work. The study is done based on cross-sectional data, the findings reported are only correlational. Not all respiratory health symptoms were diagnosed by medical doctors but only by parents. Self-reported outcomes with no scope for validation by interviewers may subject to the reporting errors. Moreover, the smoking status of family members other than parents during pregnancy and infancy were not collected. The number of cigarettes smoked per day was not included in the questionnaire, the dose of smoking could be very different from person to person and there might have been a direct dose-response relationship between the dose of smoking and respiratory outcomes^58^. Nonetheless, the present study has several strengths. A strength of our study is the ability to adjust for multiple confounders in a large sample of children. The large sample size and high response rate mean that potential confounders, such as age, sex, and geographical background, can be properly adjusted in the model.

## Conclusion

The lifetime-ever incidence rates of croup, dry night cough, frequent common cold and pneumonia among children aged 3–8 years old are 6.0%, 9.5%, 17.1%, and 32.3% respectively. The rate of maternal smoking in investigated cities was extremely small (≤1.0%) while the rate of paternal smoking was about 45%. Concerning perceived indoor tobacco odor, 25.3% of children were exposed during infancy (3.7% frequently and 21.6% sometimes) and 35.3% were exposed during childhood (6.3% frequently and 29.0% sometimes). There was a weak relationship between parental smoking and the perception of tobacco smoke odor indoors. Compared to parental smoking, indoor tobacco smoke odor was more strongly associated with respiratory outcomes. Infancy and childhood exposure (based on indoor smoke odor) were each found to be independent risk factors for respiratory outcomes; infancy exposure had a stronger effect. This research strengthens the evidence that indoor smoke exposure is a risk factor for respiratory health among children.

## References

[1] Pattenden S (2006). Parental smoking and children’s respiratory health: independent effects of prenatal and postnatal exposure. Tobacco Control.

[2] Miki K (2012). Longitudinal study of parental smoking habits and development of asthma in early childhood. Preventive Medicine.

[3] Burke H (2012). Prenatal and passive smoke exposure and incidence of asthma and wheeze: systematic review and meta-analysis. Pediatrics.

[4] Landau LI (2001). Parental smoking: asthma and wheezing illnesses in infants and children. Paediatric Respiratory Reviews.

[5] Tsai HJ, Yao TC, Chang SW, Chang WC, Huang JL (2017). Exposure to Tobacco Smoke and Childhood Rhinitis: A Population-Based Study. Journal of Allergy & Clinical Immunology.

[6] Lim S, Vos T, Bruce N (2012). ‘The burden of disease and injury attributable to 67 risk factors and risk factor clusters in 21 regions 1990-2010: a systematic analysis’. Lancet.

[7] Ahn A (2015). Secondhand Smoke Exposure and Illness Severity among Children Hospitalized with Pneumonia. Journal of Pediatrics.

[8] Miyahara R (2017). Exposure to paternal tobacco smoking increased child hospitalization for lower respiratory infections but not for other diseases in Vietnam. Scientific Reports.

[9] Carvalho RFAD (2015). Perception of parents about second hand smoke on the health of their children: an ethnographic study. Revista Paulista De Pediatria Orgao Oficial Da Sociedade De Pediatria De Sao Paulo.

[10] WHO. Protection from exposure to second-hand tobacco smoke: policy recommendations. (2007).

[11] Mishra V, Smith KR, Retherford RD (2005). Effects of Cooking Smoke and Environmental Tobacco Smoke on Acute Respiratory Infections in Young Indian Children. Population & Environment.

[12] Listed N (1984). A programme for controlling acute respiratory infections in children: Memorandum from a WHO meeting. Bull World Health Organ.

[13] Gilliland FD (2001). Effects of maternal smoking during pregnancy and environmental tobacco smoke on asthma and wheezing in children. Am J Respir Crit Care Med.

[14] Chilmonczyk BA (1993). Association between exposure to environmental tobacco smoke and exacerbations of asthma in children. New England Journal of Medicine.

[15] Jaakkola JJ (2006). Prenatal and postnatal tobacco smoke exposure and respiratory health in Russian children. Respiratory Research.

[16] Jedrychowski W, Flak E (1997). Maternal Smoking during Pregnancy and Postnatal Exposure to Environmental Tobacco Smoke as Predisposition Factors to Acute Respiratory Infections. Environmental Health Perspectives.

[17] Difranza JR, Aligne CA, Weitzman M (2004). Prenatal and postnatal environmental tobacco smoke exposure and children’s health. Pediatrics.

[18] Fuentesleonarte V (2015). Pre- and postnatal exposure to tobacco smoke and respiratory outcomes during the first year. Indoor Air.

[19] Raherison C (2007). In utero and childhood exposure to parental tobacco smoke, and allergies in schoolchildren. Respiratory Medicine.

[20] Håberg SE, Stigum H, Nystad W, Nafstad P (2007). Effects of pre- and postnatal exposure to parental smoking on early childhood respiratory health. American Journal of Epidemiology.

[21] Taylor B, Wadsworth J (1987). Maternal smoking during pregnancy and lower respiratory tract illness in early life. Archives of Disease in Childhood.

[22] Sun D (2013). China, Children, Homes, Health (CCHH). Science Bulletin.

[23] Zhang Y (2013). Ten cities cross-sectional questionnaire survey of children asthma and other allergies in China. Science Bulletin.

[24] Pearce N (1993). Self-reported prevalence of asthma symptoms in children in Australia, England, Germany and New Zealand: an international comparison using the ISAAC protocol. Eur.respir.j.

[25] Bornehag CG, Sundell J, Sigsgaard T (2004). Dampness in buildings and health (DBH): Report from an ongoing epidemiological investigation on the association between indoor environmental factors and health effects among children in Sweden. Indoor Air.

[26] Naydenov K (2008). The association of pet keeping at home with symptoms in airways, nose and skin among Bulgarian children. Pediatric Allergy & Immunology Official Publication of the European Society of Pediatric Allergy & Immunology.

[27] Cook DG, Strachan DP (1997). Health effects of passive smoking. 3. Parental smoking and prevalence of respiratory symptoms and asthma in school age children. Thorax.

[28] Stoddard JJ, Miller T (1995). Impact of parental smoking on the prevalence of wheezing respiratory illness in children. American Journal of Epidemiology.

[29] Vardavas CI (2016). The independent role of prenatal and postnatal exposure to active and passive smoking on the development of early wheeze in children. European Respiratory Journal.

[30] Everard M (2009). Acute bronchiolitis and croup. Pediatric Clinics of North America.

[31] Bjornson CL, Johnson DW (2013). Croup in children. CMAJ: Canadian Medical Association journal=journal de l’Association medicale canadienne.

[32] Rajapaksa S, Starr M (2010). Croup - assessment and management. Australian Family Physician.

[33] Pruikkonen H, Dunder T, Renko M, Pokka T, Uhari M (2009). Risk factors for croup in children with recurrent respiratory infections: a case-control study. Paediatric & Perinatal Epidemiology.

[34] Chang AB, Berkowitz RG (2010). Cough in the Pediatric Population. Otolaryngol Clin North Am.

[35] Hall CB, Wakefield D, Rowe TM, Carlisle PS, Cloutier MM (2001). Diagnosing pediatric asthma: validating the Easy Breathing Survey. Journal of Pediatrics.

[36] Gehring U (2002). Traffic-related air pollution and respiratory health during the first 2yrs of life. European Respiratory Journal.

[37] Qian H, Zheng X, Zhang M, Weschler L, Sundell J (2016). Associations between Parents’ Perceived Air Quality in Homes and Health among Children in Nanjing, China. PloS one.

[38] Fuentes-Leonarte V, Tenías JM, Ballester F (2009). Levels of pollutants in indoor air and respiratory health in preschool children: a systematic review. Pediatr Pulmonol.

[39] Roda C, Guihenneuc-Jouyaux C, Momas I (2013). Environmental triggers of nocturnal dry cough in infancy: New insights about chronic domestic exposure to formaldehyde in the PARIS birth cohort. Environmental Research.

[40] Sucharew H (2010). Exposure to traffic exhaust and night cough during early childhood: the CCAAPS birth cohort. Pediatric Allergy & Immunology.

[41] Linehan M (2008). Prevalence of respiratory symptoms, features of asthma, and characteristics associated with respiratory disease, in 6–11 year olds in Manchester. Primary Care Respiratory Journal.

[42] Lorber B (1996). The common cold. Journal of General Internal Medicine.

[43] Simasek M, Blandino DA (2007). Treatment of the common cold. American Family Physician.

[44] Dan N (2017). Common cold among pre-school children in China - associations with ambient PM 10 and dampness, mould, cats, dogs, rats and cockroaches in the home environment. Environment International.

[45] WHO. Pneumonia fact sheet No 331. Available at, http://who.int/mediacentre/factsheets/fs331/en/ (accessed. 2016).

[46] Wardlaw T, Johansson EW, Hodge M (2006). Pneumonia: the forgotten killer of children. New York New York Unicef Sep.

[47] Omiyefa S, Osoba R (2012). 110 Environmental Tobacco Smoke as a Risk Factor to Increasing Respiratory Childhood Infection and Pneumonia in South-West region Nigeria. European Journal of Cancer.

[48] Singh V (2005). The burden of pneumonia in children: an Asian perspective. Paediatric Respiratory Reviews.

[49] Zhuge Y (2018). Residential risk factors for childhood pneumonia: A cross-sectional study in eight cities of China. Environment international.

[50] Zheng X (2013). Home risk factors for childhood pneumonia in Nanjing, China. Chinese Science Bulletin.

[51] Chen Y, Li WX, Yu SZ, Qian WH (1988). Chang-Ning epidemiological study of children’s health: I: Passive smoking and children’s respiratory diseases. International Journal of Epidemiology.

[52] Hajnal BL (1999). Effect of environmental tobacco smoke exposure on respiratory symptoms in children. SCARPOL Team. Swiss Study on Childhood Allergy and Respiratory Symptoms with Respect to Air Pollution, Climate and Pollen. Schweizerische Medizinische Wochenschrift.

[53] Mannino DM, Moorman JE, Kingsley B, Rose D, Repace J (2001). Health effects related to environmental tobacco smoke exposure in children in the United States: data from the Third National Health and Nutrition Examination Survey. Journal of Research in Science Teaching.

[54] Le DR, Myer L, Nicol MP, Zar HJ (2015). Incidence and severity of childhood pneumonia in the first year of life in a South African birth cohort: the Drakenstein Child Health Study. Lancet Global Health.

[55] Talati A, Wickramaratne PJ, Wesselhoeft R, Weissman MM (2017). Prenatal tobacco exposure, birthweight, and offspring psychopathology. Psychiatry Research.

[56] Tettamanti G, Ljung R, Mathiesen T, Schwartzbaum J, Feychting M (2015). Maternal smoking during pregnancy and the risk of childhood brain tumors: Results from a Swedish cohort study. Cancer Epidemiology.

[57] WHO. WHO global report on trends in prevalence of tobacco smoking 2015. (2015).

[58] Dagvadorj A (2016). Hospitalization risk factors for children’s lower respiratory tract infection: A population-based, cross-sectional study in Mongolia. Scientific Reports.

